# Verification of Composite Beam Theory with Finite Element Model for Pretensioned Concrete Members with Prestressing FRP Tendons

**DOI:** 10.3390/ma16196376

**Published:** 2023-09-24

**Authors:** Xin Sha, James S. Davidson

**Affiliations:** 1 Department of Civil and Environmental Engineering, Auburn University, Auburn, AL 36849, USA; xzs0010@auburn.edu; 2 Faculty of Civil Engineering and Mechanics, Jiangsu University, Zhenjiang 212013, China

**Keywords:** composite beam theory, prestressed FRP tendons, transfer length, reinforced concrete, finite element analysis (FEA), friction coefficient, bond–slip relationship

## Abstract

Composite beam theory was previously developed to establish an analytical solution for determining the transfer length of prestressed fiber-reinforced polymers (FRP) tendons in pretensioned concrete members. In the present study, a novel finite element (FE) modeling approach is proposed to provide further verification of the developed analytical method. The present FE model takes into account the friction coefficients obtained from pull-out tests on the FRP tendons and prestressed concrete members. Convergence analysis of two numerical simulations with different mesh densities is carried out as well. The results demonstrated that the transfer length predicted by the fine FE model with a friction coefficient of α = 0.3 for high pretension is in good agreement with the measured values and the analytical solutions. The consistency between the analytical solution and FE simulation not only further proves the reliability of composite beam theory but also demonstrates the importance of the bond–slip relationship in predicting the transfer length of pretensioned concrete members prestressed with FRP tendons.

## 1. Introduction

Along with the strikingly rapid development of composite materials in the field of civil engineering, the applications of fiber-reinforced polymers (FRP) in strengthening concrete structures have attracted more and more attention. CFRP has higher mechanical properties, excellent fatigue resistance, corrosion resistance, and creep resistance. BFRP and GFRP have relatively low prices and wide sources. However, their long-term performances in service environments may degrade [1,2,3]. The applications of FRP can be divided into the form of FRP tendons as internal reinforcements or FRP laminates as externally bonded reinforcements. In the previous decades, extensive analytical and experimental studies have been conducted on the local bond–slip relationship of concrete flexural members reinforced by FRP tendons [4,5,6,7,8,9,10,11] or concrete members strengthened by externally bonded FRP laminates [12,13,14,15,16,17,18,19,20,21,22]. It was found that a sound understanding of the bond behavior between FRP reinforcement, and the concrete substrate played a major role in the development of design guidelines and the performance evaluation of FRP-strengthening concrete members. Therefore, a reliable and rigorous analytical model based on the innovative partial composite action, taking into account the corresponding bond characteristics, is essential to accurately assess the mechanical properties of strengthening or retrofitting concrete structures using FRP. A new method [23] was previously developed for determining the transfer length of pretensioned concrete with prestressing FRP tendons, which was solved with closed-form solutions using composite beam theory associated with considering the local bond–slip relationship between FRP tendons and concrete. In the presented paper, finite element modeling of pretensioned concrete members with prestressing FRP tendons is proposed in order to provide further verification of the developed analytical methodology.

Composite beam theory, proposed by Granholm [24] in 1949 and Newmark et al. [25] in 1951, was initially used to solve the cases of nailed timber structures and T-beams consisting of a rolled steel I-beam and a concrete slab, respectively. In terms of partially composite action, the theoretical analysis was developed for the member consisting of two separate elements connected by discrete connectors. Furthermore, the influence of relative displacement between the two elements, i.e., the effect of slip, was fully considered. From this perspective, therefore, composite beam theory is not limited to the types of structures mentioned above but is instead devoted to a wide range of structures comprised of two or more interconnected elements under reasonable assumptions. For example, Bai and Davidson [26] implemented a rigorous analysis of foam-insulated concrete sandwich panels in which structural deflection was discomposed into two components, shear and flexural. The structural behavior was taken into account as partially composite in terms of composite beam theory. Sha and Davidson [23,27] provided closed-form solutions using composite beam theory for determining the transfer length of pretensioned concrete members strengthened by FRP tendons as well as predicted the interfacial stress in concrete beams with externally bonded FRP laminates. Through the research on the developmental course of composite beam theory [23,25,26], it has been observed that theoretical methods were mainly verified against the existing experimental data from the literature. However, in order to comprehensively evaluate the accuracy and reliability of the developed method, as a supplementary verification, finite element analyses (FEA) are an effective methodology that can be employed to compare with theoretical solutions.

As an important numerical technique, FEA has been widely used to study the behavior of prestressed concrete beams [28,29,30]. Most research has focused on pretensioned concrete members with prestressing steel strands; only a very limited number of FE models are specifically available for pretensioned concrete with prestressing FRP tendons. The main reason for the lack of in-depth FE research in this field is the challenging nature of the interaction between FRP tendons and the concrete matrix. Hence, this paper establishes a three-dimensional FE model that simulates prestressing FRP tendons in which the transfer length is determined. In addition, the different friction coefficients between FRP tendons and the surrounding concrete obtained by experimental studies [31] are fully considered to improve the accuracy of FE modeling approaches.

One of the most compelling advantages of FEA over other analytical solutions is that a simulation associated with fewer assumptions may be closer to the corresponding experimental outcomes. In addition, visualizations of the pre- and postprocessing of FEA can help engineers easily find vulnerabilities in the design. Despite some obvious advantages, mesh convergence is a critical issue that must be taken into account in the process of developing FE models. In this work, a comparative study is conducted between numerical simulation with fine and coarse meshes to illustrate the effect of mesh density on convergence. Another noteworthy point is that the concrete model used for numerical simulation is based on the linear elastic assumption. Although the concrete damaged plasticity (CDP) model from Abaqus [32] has often been used to simulate the nonlinear behavior of concrete in other studies, the strains associated with the present paper are assumed to be in a range that essentially has a linear and brittle stress–strain relationship in compression. Furthermore, pretensioned members are designed for zero tension in the concrete under service load conditions through Rabbat et al.’s [33] tests. The main focus of this study is to determine the transfer length at the serviceability state level in which concrete has not yet cracked, and, therefore, it is reasonable to assume that the concrete is within the linear-elastic range.

In order to provide convenient use in engineering practice, the key technical challenge of this study is to develop a general form of the governing equations specifically for FRP-strengthening concrete members in terms of composite beam theory. Taking account of the empirical bond–slip relationship between FRP tendons and the concrete matrix, governing differential equations are derived in terms of the equilibrium of axial force acting on each element as well as the balance of the overall bending moment. Using the FEA commercial software Abaqus [32], a comparison of the transfer length of prestressing FRP tendons in pretensioned concrete with those obtained by using composite beam theory is conducted. The present FE model has been established with consideration of the friction coefficient from the experimental study on the FRP tendons and prestressed concrete members. Additionally, different mesh densities are compared for the convergence analysis. As a result, a satisfactory agreement has been reached between the theoretical solutions and FEA responses, which further demonstrates the feasibility and effectiveness of the developed composite beam theory.

## 2. Background of the Bond Mechanism

Understanding the nature of bond behavior plays a critical role in assessing how the prestress force is transferred from the prestressing FRP tendons to the concrete. A large amount of research [28,30,34,35] indicates that the chemical adhesive, friction, and mechanical interlocking could explain the interaction between prestressing tendons and concrete. Chemical adhesives only affect the bond strength in the minimal slip range. With the increase of slip, friction and mechanical interlocking play roles in the bond strength when the adhesive bond gradually decreases. For prestressed tendons with a rough surface, such as seven-wire strands, ribbed bars, and deformed rebars, the mechanical action of the helical outer wire of a strand bearing against the surrounding concrete matrix is referred to as mechanical interlocking. It should be noted, however, that, although the contribution of mechanical interlocking to bond strength is important, it is still not the key factor. This is because the rough surface of surrounding concrete that is in contact with prestressing tendons will eventually be sheared off due to the mechanical interlock action if the pretensioned structure has sufficient confinement. However, that does not seem to be occurring [35]. In other words, this would imply that friction known as the “wedge effect” dominates the interaction between prestressed tendons and concrete.

**Figure 1:**
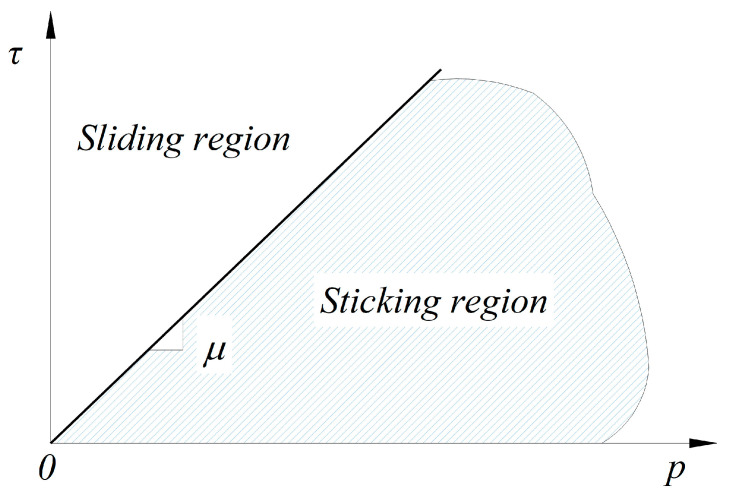
Coulomb friction model [32].

Friction can be defined as a relationship that is responsible for transmitting the shear and normal forces between contacting bodies, i.e., prestressing tendons and the surrounding concrete matrix. According to the commercial FE program Abaqus [32], friction behavior is generally analyzed using the base form of the Coulomb friction model in which the critical shear stress is given by the following expression:

(1) $$\tau_{crit}\;=\;\mu \;p,$$

where $\tau_{crit}$ is the critical shear stress, p is the contact pressure, and μ is the friction coefficient. In the Coulomb friction model shown in Figure 1, the shear stresses between two contacting surfaces, τ ≤ μ p, is the case in which the two contacting bodies are in a state of sticking before sliding occurs, and τ > μ p is when shear stresses exceed a certain magnitude defined as the critical shear stress $\tau_{crit}$, which refers to the transition from sticking to slipping along the interface of contacting bodies. The slope of the function, the friction coefficient μ, is in the range of 0.3 to 0.7 according to most research literature [29,35]. However, AASHTO [36] reports that the value of the friction coefficient increases from approximately 0.6 to 1.4, depending on the concrete surface conditions and the shape of the reinforcement. Thus, it can be seen that some inconsistencies exist between the specification and the literature used to explain the bond behavior between concrete members and the reinforcement, which directly affects the reliability of the analysis results based on the value of the friction coefficient.

It is worth mentioning that the current work using FEM to estimate the transfer length is based on the friction coefficient specifically for FRP tendons in pretensioned prestressed concrete members. Previous studies on finite element analysis [28,29,30] of pretensioned concrete members used the value of the friction coefficient recommended by the specification to address the bond behavior, which is suitable for steel reinforcement as a prestressed strand. However, when FRP tendons are considered, it is necessary to redefine the friction coefficient through the available experimental data. Khin et al. [31] carried out pull-out tests of Vinylon and Carbon FRP tendons with cement mortar and confined by highly expansive material (HEM). During the test, the bond stress versus confining pressure for specimens was recorded using high-precision pressure transducers to determine the friction coefficient of FRP tendons from the slope of the curve. These values from Khin et al. [31] are listed in Table 1 and used as the friction coefficient in the presented FE model.

For another description of the bond-behavior model, the local bond stress–slip relationship τ = τ (s) was used in the analytical model [23] developed by using composite beam theory for determining the transfer length for FRP tendons in prestressed concrete. The results of pullout tests [4,5,7,37,38,39] show that the local bond stress as a function of slip depends on a variety of factors, including concrete strength, the roughness of the reinforcement surface, concrete cover, bar diameter, and epoxy resin properties. Over the years, numerous existing models of the bond stress τ and slip s have been proposed to evaluate the bond performance that is established on the basis of the nonlinear local bond stress–slip relationship τ = τ (s) between concrete and the reinforcement. Three well-known models have been developed for steel and FRP tendons, namely the Bertero–Eligehausen–Popov (BEP) model [4], the modified Bertero–Eligehausen–Popov (mBEP) model, and the Cosenza–Manfredi–Realfonzo (CMR) model [5]. The BEP model is defined by Equation (2), which is adopted in CEB-FIP Model Code 90 [37]:

(2) $$\tau \;=\;\tau_{0}\;{\left(\frac{s}{s_{0}}\right)}^{\alpha },$$

where $\tau_{0}$ is the maximum shear stress, $s_{0}$ is the slip corresponding to $\tau_{0}$, and α is the coefficient of 0.4 that is available for the case of steel [37]. Considering different requirements in the engineering analysis process, the mBEP and CMR expressions were proposed as the bond stress–slip alternative analytical models given by the following Equations (3) and (4), respectively.

(3) $$\tau \;=\;C\;s^{\alpha }\;\left(1\;-\;\frac{s}{\bar{s}}\right),$$

(4) $$\tau \;=\;\tau_{m}\;{\left[1\;-\;\exp\;(-\;\frac{s}{s_{r}})\right]}^{\beta },$$

**Figure 2:**
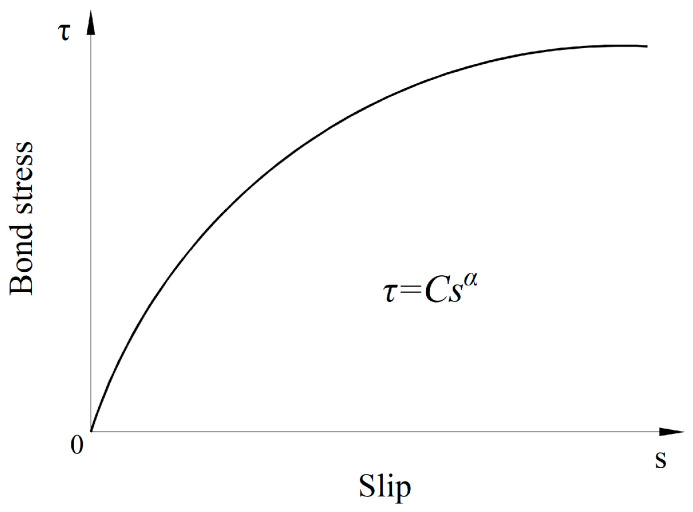
BEP model of bond–slip relationship [7].

For the mBEP model, $\tau_{0}$ is rewritten by C and, assuming $s_{0}\;=\;1\;\mathrm{mm}$ from the BEP expression, $\bar{s}$ is the slip related to τ = 0. The expression of CMR, $\tau_{m}$ is the peak bond stress, and the unknown parameters $s_{r}$ and β are determined by the curve fitting of the experimental data. More detailed reviews of these analytical models for the curve τ − s can be found in the literature [4,5,7,23]. In previous work by Sha and Davidson [23], the BEP expression with calibrated parameters of C and α from Focacci et al. [7] was used as the constitutive bond–slip definition between the FRP tendons and concrete, as illustrated in Figure 2. The latter two models, i.e., the mBEP and CMR expressions, are equivalent to the BEP expression in the case of structural analyses in which the slip is sufficiently small.

Thick-wall cylinder theory [29] depends on the Coulomb friction model to perform the analysis on prestress transfer in pretensioned concrete members. The concrete is conceived as a hollow cylinder in which the inner diameter is equal to that of prestressed tendons and the outer diameter is the distance across the short side (diameter) of the component. Accordingly, the estimation of bond behavior relies on the radial compressive stress as well as deformation compatibility conditions of the interface between prestressed tendons and the surrounding concrete. Based on extensive experimental and analytical investigations [5,39,40], many researchers nevertheless point out that the confinement pressure has a small effect on the bond strength between the reinforcement (steel or FRP) and the surrounding concrete for the situation in which the outer surface of the reinforcement bar is a spiral. However, the bond resistance strongly depends on the confined stress known as radial compressive stress in other cases such as smooth rods. Different from the thick-wall cylinder theory, the nonlinear bond stress–slip relationship is taken into account for deriving the governing differential equations using composite beam theory developed herein for analyzing the behavior of pretensioned concrete members with prestressing FRP tendons. Consequently, in addition to further verifying the previous work of predicting transfer length for prestressing FRP tendons by means of the developed FE model, the second aim of the current study is to prove the superiority of composite beam theory considering the slip effect through the comparative studies between the analytical and numerical results.

## 3. Analytical Solution for FRP-Strengthening Concrete Members

### 3.1. General Approach of Composite Beam Theory

The following assumptions and limitations are specifically proposed for FRP-strengthening concrete members using the general approach of composite beam theory:

(1) Linear elastic constitutive behavior and small displacement are applied to each component in the developed analytical models;
(2) FRP’s bending resistance is negligible compared to that provided by the concrete member;
(3) Slender beams are considered and therefore Euler–Bernoulli beam theory can be applied to each component of a composite structure, and, therefore, the shear deformation is neglected through the cross section of the concrete beam and FRP components, respectively.

#### 3.1.1. Axial Force Equilibrium

**Figure 3:**
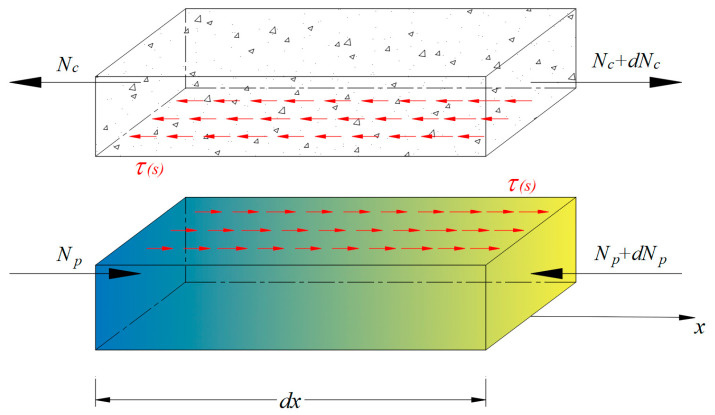
Differential element of the composite beam.

A differential element of the composite beam is composed of the concrete and FRP, as illustrated in Figure 3; the upper part represents concrete and the lower part is FRP. Axial forces acting on the cross sections of two separate infinitesimal elements are transmitted as internal forces through bond stress τ that is a function of slip. As a result, equilibrium can be established in Equation (5):

(5) $$\tau \;C_{F}\;dx\;=\;dN_{c}\;=\;dN_{p},$$

in which $N_{c}$ and $N_{p}$ denote the resultant axial force acting on the cross section of the concrete and FRP, respectively, and $C_{F}$ represents the contact length of the interface between FRP and the surrounding concrete in the transverse direction. For instance, $C_{F}$ can refer to the total circumferences of FRP tendons in prestressed concrete members. In the case of a concrete beam strengthened by externally bonded FRP laminates, it is the width of FRP laminates. Substituting $N_{c}\;=\;\sigma_{c}\;A_{c}$ and $N_{p}\;=\;\sigma_{p}\;A_{p}$ into Equation (5) results in:

(6) $$\tau \;C_{F}\;=\;{\left(A_{c}\;\sigma_{c}\;\right)}^{\prime }\;=\;{\left(A_{p}\;\sigma_{p}\;\right)}^{\prime },$$

in which $A_{c}$ is the cross section area of concrete, $\sigma_{c}$ is the concrete stress due to axial force, $A_{p}$ is the cross-section area of FRP, and $\sigma_{p}$ is the FRP stress due to axial force. Accordingly, σ = E ε applies to both the concrete and FRP elements due to the assumption of the linear elastic constitutive behavior. Equation (6) then becomes:

(7) $$\tau \;C_{F}\;=\;A_{c}\;E_{c}\;\varepsilon_{c}{}^{\prime }\;=\;A_{c}\;E_{c}\;s_{c}{}^{\prime \prime },$$

(8) $$\tau \;C_{F}\;=\;A_{p}\;E_{p}\;\varepsilon_{p}{}^{\prime }\;=\;A_{p}\;E_{p}\;s_{p}{}^{\prime \prime },$$

where $E_{c}$ and $E_{p}$ refer to the modulus of elasticity of the concrete and FRP, respectively. Since ${s^{\prime }}_{c}$ is the first derivative of the concrete displacement $s_{c}$ due to the axial force, the concrete axial strain is $\varepsilon_{c}\;=\;{s^{\prime }}_{c}$. Similarly, $s_{p}$ represents the FRP displacement due to the axial force, $\varepsilon_{p}\;=\;{s^{\prime }}_{p}$ corresponds to FRP strains.

As mentioned above, the analysis of the behavior of FRP-strengthening concrete members is based on partial composite action. To some extent, this means that relative movement between FRP and the surrounding concrete, namely slip s, is permitted.

(9) $$s\;=\;s_{1}\;+\;s_{2}\;+\;s_{3},$$

(10) $$s_{2}\;=\;s_{c}\;+\;s_{p}.$$

Thus, Equation (9) indicates that the slip s can be divided into three components: the slip $s_{1}$ due to bending illustrated later, the relative displacement $s_{2}$ between the concrete and FRP due to the axial force, as expressed by Equation (10), and the slip $s_{3}\;=\;\int \varepsilon_{is}\;dx$ resulting from prestressing tendon retraction, where $\varepsilon_{is}$ is the strain caused by prestress before transmission. In the case of a concrete beam strengthened by externally bonded FRP laminates, $s_{3}\;=\;0$.

In terms of the τ = τ (s) relationship obtained by experimental studies on FRP reinforcement in concrete, combined with Equation (10), the new equilibriums are established by Equations (11) and (12):

(11) $$\tau \;(s)\;C_{F}\;=\;A_{c}\;E_{c}\;\frac{{s^{\prime \prime }}_{2}}{\left(1\;+\;\frac{A_{c}\;E_{c}}{A_{p}\;E_{p}}\right)},$$

(12) $$\tau \;(s)\;C_{F}\;=\;A_{p}\;E_{p}\;\frac{{s^{\prime \prime }}_{2}}{\left(1\;+\;\frac{A_{p}\;E_{p}}{A_{c}\;E_{c}}\right)}.$$

As a result, the governing equation relevant to the axial force is generated in the form:

(13) $$\tau \;(s)\;=\;\zeta \;{s^{\prime \prime }}_{2},$$

in which $\zeta \;=\;\frac{A_{c}\;E_{c}\;A_{p}\;E_{p}}{C_{F}\;\left(A_{c}\;E_{c}\;+\;A_{p}\;E_{p}\right)}$.

#### 3.1.2. Bending Moment Equilibrium

**Figure 4:**
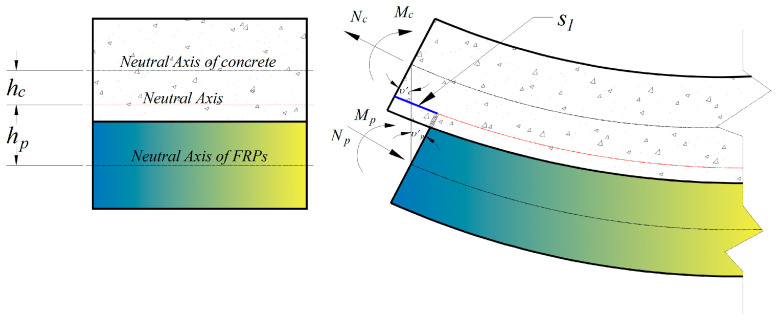
The slip $s_{1}$ due to bending moment.

The known external moment $M_{ex}$ is balanced by the internal moment and axial force acting on the cross section of each element, as expressed in the following:

(14) $$M_{ex}\;=\;M_{c}\;+\;M_{p}\;+\;N_{c}\;h_{c}\;+\;N_{p}\;h_{p},$$

where the internal bending moment $M_{c}\;=\;-\;E_{c}\;I_{c}\;{\upsilon^{\prime \prime }}_{c}$ in the concrete beam with the second derivative of the deflection ${\upsilon^{\prime \prime }}_{c}$ and the moment of inertia $I_{c}$. Bending resistance from the FRP component is ignored, $M_{p}\;=\;0$. As demonstrated in Figure 4, $h_{c}$ is the distance from the concrete-beam centroid to the neutral axis, and, similarly, $h_{p}$ corresponds to FRP. Combining Equations (7) and (8), the internal axial forces in the concrete and FRP are given by:

(15) $$N_{c}\;=\;N_{p}\;=\;\zeta \;C_{F}\;{s^{\prime }}_{2}.$$

Furthermore, it can be observed that the slip $s_{1}$ is associated with the neutral axis and the slope angle of each part when the bending moment is occurring. The slip $s_{1}$ can be written in the general form:

(16) $$s_{1}\;=\;h_{c}\;{\upsilon^{\prime }}_{c}\;+\;h_{p}\;{\upsilon^{\prime }}_{p},$$

where ${\upsilon^{\prime }}_{c}$ denotes the slope angle of concrete, ${\upsilon^{\prime }}_{p}$ relevant to that of FRP. Considering that concrete flexural members are reinforced by FRP tendons, the slip $s_{1}$, in this case, can be expressed as follows:

(17) $$s_{1}\;=\;e\;\upsilon^{\prime }.$$

The reason for this change is that the structural behavior is limited to elastic and small displacement, the constitutive relation is assumed to be linear, and each component of the composite beam has the same deflection $\upsilon_{c}\;=\;\upsilon_{p}\;=\;\upsilon$. In the meantime, it will be readily understood that the eccentricity is equal to the distance from the neutral axis of the concrete beam to the FRP′, i.e., $e\;=h_{c}\;+\;h_{p}$.

Substituting Equation (15) into Equation (14) results in the expression for the bending moment as follows:

(18) $$M_{ex}\;=\;-E_{c}\;I_{c}\;{\upsilon^{\prime \prime }}_{c}\;+\;\zeta \;C_{F}\;{s^{\prime }}_{2}\;\left(h_{c}\;+\;h_{p}\right).$$

Consequently, plugging $s_{2}\;=\;s-s_{1}\;-\;s_{3}$ into the differential equations, Equations (13) and (18), and simplifying the set of governing equations that are specifically developed for FRP-strengthening concrete members using composite beam theory can be given by:

(19) $$\left\{\begin{array}{l} s^{\prime \prime }\;-\;\frac{\tau (s)}{\zeta }\;=\;h_{c}\;{\upsilon^{\prime \prime \prime }}_{c}\;+\;h_{p}\;{\upsilon^{\prime \prime \prime }}_{p}\\ \frac{{\upsilon^{\prime \prime }}_{c}\;}{\alpha_{s}}\;+\;h_{p}\;{\upsilon^{\prime \prime }}_{p}\;-\;s^{\prime }\;=\;-\;\frac{M_{ex}}{D_{s}}\;-\;\varepsilon_{is} \end{array}\right.,$$

in which the new symbols are introduced for simplicity:

(20) $$D_{s}\;=\;\zeta \;C_{F}\;(h_{c}\;+\;h_{p}),$$

(21) $$\alpha_{s}\;=\;\frac{D_{s}}{E_{c}\;I_{c}\;+\;D_{s}\;h_{c}},$$

where ${\upsilon^{\prime \prime \prime }}_{c}$ represents the third derivative of the deflection of concrete, ${\upsilon^{\prime \prime \prime }}_{p}$ relevant to that of FRP. According to the general form of the governing equation, Equation (19), it can be observed that the bond–slip relationship τ = τ (s) corresponding to various forms of FRP-strengthening concrete structures would be taken into account. For the application of prestressed concrete with FRP tendons, the governing equations can be generated by combining the relationship of bond–slip, i.e., $\tau (s)\;=\;8.847\;s^{0.337}$ [7], in metric (SI) units [23]:

(22) $$\left\{\begin{array}{l} s^{\prime \prime }\;-\;\frac{8.847}{\zeta }\;s^{0.337}\;=\;e\;\upsilon^{\prime \prime \prime }\\ \upsilon^{\prime \prime }\;-\;\frac{T_{s}{}^{2}}{e}\;s^{\prime }\;=\;-\;\frac{M_{ex}}{K_{s}}\;-\;\frac{T_{s}{}^{2}}{e}\;\varepsilon_{is} \end{array}\right.$$

where $\upsilon^{\prime \prime \prime }$ denotes the third derivative of the deflection of the composite beam,

(23) $$K_{s}\;=\;E_{c}\;I_{c}\;+\;e^{2}\;\zeta \;C_{F},$$

(24) $$T_{s}^{2}\;=\;\frac{e^{2}\;\zeta \;C_{F}}{K_{s}}.$$

### 3.2. Predictions of Transfer Length for Prestressed FRP Tendons Application

**Figure 5:**
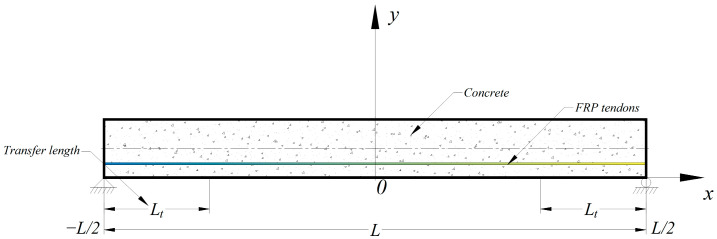
The coordinate system of the pretensioned concrete with prestressed FRP tendons.

It is critical to estimate the transfer length of prestressed concrete beams, not only because it affects the bending and shear strength of the structure, but also because it is valuable for designers to understand it for structural detailing. The effective prestressing force is transferred from the prestressed tendons to the concrete in the transfer zone, in which the distance is related to the transfer length. The conventional experimental investigation used to measure the transfer length mainly depends on the strain change in the concrete or the prestressed tendons before and after release. The determination of transfer length is implemented in terms of the strain distribution along the length of the beam using the 95% Average Maximum Strain (AMS) method. For the analytical solution, the proposed model relied upon a novel and theoretically pure composite beam theory developed specifically for pretensioned prestressed concrete members with FRP tendons, the details of which were provided in the previous work [23]. Based on the boundary condition of a simply supported beam without any external loading, as shown in Figure 5, correspondingly, the closed form solution with α = 0.337 for the governing equation, Equation (22), is given by the following, in metric (SI) units [23]:

(25) s(x) = 0 ,                                 0 ≤ x ≤ L2 − LtA (x − L2 + Lt)B ,  otherwise

in which

(26) $$L_{t}\;=\;{\left[\frac{\varepsilon_{is}\;\left(1\;+\;\alpha \right)\;{\left(0.5\;\varepsilon_{is}\right)}^{-\;\alpha }}{\psi \;{\left(1\;-\;\alpha \right)}^{1\;+\;\alpha }}\right]}^{\frac{1}{1\;+\;\alpha }}$$

(27) $$A\;=\;e^{\frac{\ln\;\frac{2\;\left(1\;+\;\alpha \right)}{\psi \;{\left(\alpha \;-\;1\right)}^{2}}}{\alpha \;-\;1}},$$

(28) $$B\;=\;\frac{2}{1\;-\;\alpha },$$

(29) $$\psi \;=\;\frac{8.847}{\zeta \;\left(1\;-\;T_{s}^{2}\right)}.$$

Transfer length $L_{t}$, expressed by Equation (26), is derived by taking advantage of the type of piecewise function for the slip s (Equation (25)), considering the local bond–slip relationship [7] resulting from the average behavior of FRP tendons with diameters of 6.4 mm, 9.5 mm, 12.7 mm, and 15.9 mm. Previously, it has been validated through the comparison between the prediction of the strain profile and the available experimental data from the literature.

## 4. Numerical Implementation with Finite Element Modeling

### 4.1. Finite Element Modeling of Pretensioned RC Beams with FRP Tendons

**Figure 6:**
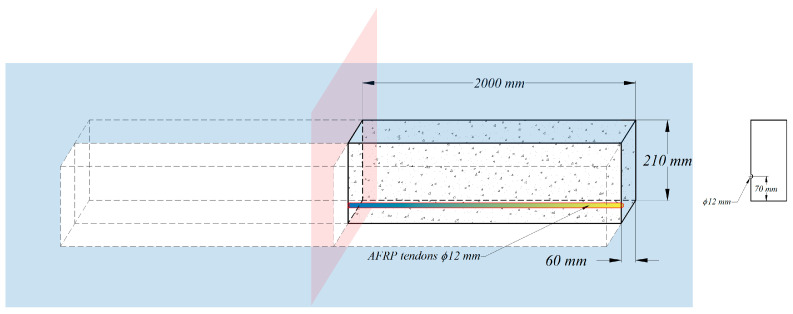
Geometric details of ¼ of the beam using double-symmetry conditions in Abaqus.

Experimental studies on the transmission of prestressing force conducted by Nanni et al. [41] are utilized herein to develop an FE model using the commercial software Abaqus [32]. As a result, the strain profiles are provided for the determination of transfer length and further verify the general approach of composite beam theory. The specimen is a simply supported beam with the span of 4000 mm and the rectangular cross section of 210 mm  ×  120 mm. The pretensioned concrete beam prestressed with a single AFRP tendon is modeled using two levels of 50% and 100% initial prestress force. In order to simulate the prestressing process, the prestresses of 698 MPa and 349 MPa are applied to the prestressing tendon in the initial step, respectively. Taking advantage of double-symmetry conditions, a quarter of pretensioned RC beams with FRP tendons modeled in the simulation are given as shown in Figure 6. As explained in the previous section, both concrete and FRP tendons are modeled as linear elastic, isotropic materials with parameters reported in Table 2.

**Figure 7:**
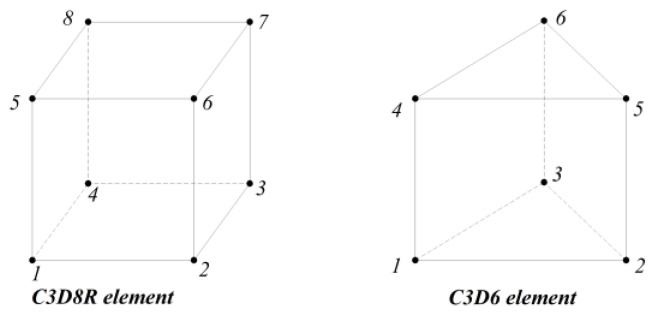
Geometric characteristics of elements used for concrete and FRP tendons.

C3D8R with eight-node linear brick is employed for modeling the concrete in Abaqus, which is a reduced integration element with hourglass control. The C3D6 element, which is a six-node linear triangular prism, is used for the FRP tendons. The geometries of these two elements are illustrated in Figure 7.

**Figure 8:**
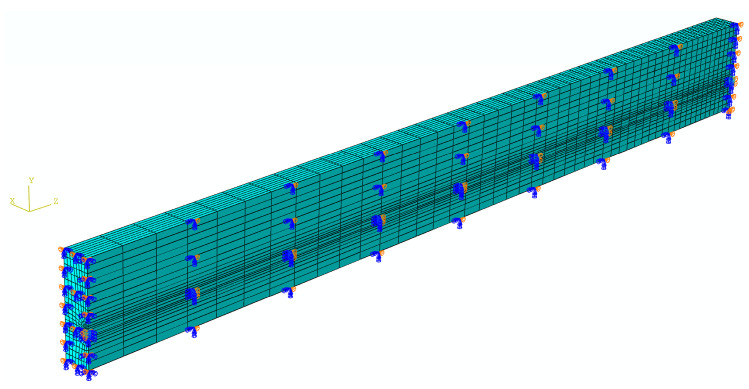
Finite element model of a pretensioned concrete beam with boundary conditions.

In order to optimize the computing time and the results precision, the mesh around the FRP tendons is very refined, and the mesh density increases as the distance from the midspan increases. The quarter symmetry FE model of a pretensioned concrete beam with boundary conditions is represented in Figure 8. As can be seen, the symmetry restraints on the Y–Z and X–Y symmetry planes are U1 = UR2 = UR3 = 0 and U3 = UR1 = UR2 = 0, respectively. Meanwhile, a support condition of UY = 0 is applied to the end of the beam.

The interaction between the concrete and FRP tendons modeled by FE consists of three parts: tangential behavior, normal behavior, and cohesive behavior. According to the concept of the Coulomb friction model, as previously described, the tangential behavior between two contacting surfaces is defined in accordance with four different friction coefficients μ listed in Table 1 from Khin et al. [31]. The reason for this is to explain variable bond stress along the beam in the region of transfer length. Normal behavior is modeled using “hard” contact as the contact pressure–overclosure relationship, which prevents penetration of the concrete into FRP tendons in the FE simulation of bond behavior. Another consideration is to prevent the transmission of tensile stress through the interface between the FRP tendons and concrete. The cohesive behavior adopted in the present FEA is due to the slip at the interface. Moreover, since the purpose of this research is to predict the transfer length at the serviceability state using FEA and to further verify the previously proposed composite beam theory, creep, and shrinkage are not considered.

### 4.2. Convergence Analysis and Verification of FE Model

**Figure 9:**
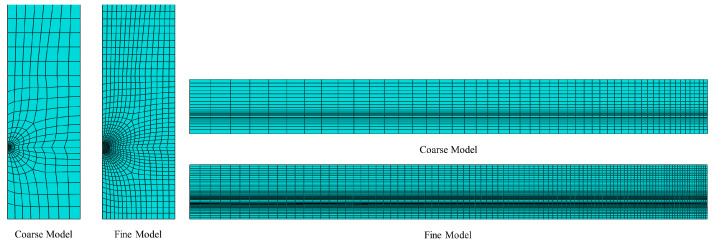
Mesh density for both coarse model and fine model.

**Figure 10:**
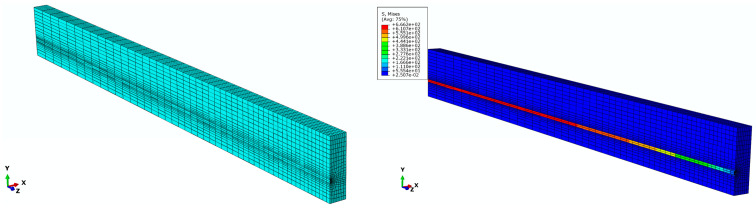
Finite element model with coarse mesh.

**Figure 11:**
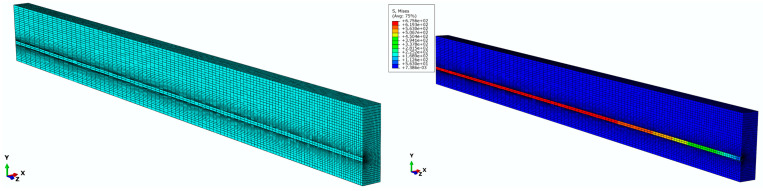
Finite element model with fine mesh.

In order to investigate the dependence of nonlinear solutions obtained from the proposed FE model on the mesh size, two models with different mesh densities are simulated in Abaqus. By means of the solution technique of full Newton and automatic control of the time increment, the mesh convergence study is performed on the three-dimensional FE model of a pretensioned concrete beam using coarse mesh and fine mesh, respectively. The cross sectional and longitudinal views corresponding to both the fine and coarse models are shown in Figure 9. Two models associated with coarse and fine are meshed by defining the number of elements along the selected edges, where the number of elements defined on each edge of the fine model is twice as those applied in the coarse model. For the coarse mesh shown in Figure 10, the FE model is composed of 10,000 hexahedral elements of type C3D8R and 800 wedge elements of type C3D6. A similar meshing method is correspondingly given in the fine model, which is made up of 77500 hexahedral elements for concrete as well as 3200 wedge elements for FRP tendons, as shown in Figure 11. Eventually, in order to judge the convergence of the mesh refinement solution, Figure 10 and Figure 11 are compared. It is worth noting that the result has a very close agreement with a 1% difference between the maximum von Mises stress that occurs at the midspan of FRP tendons.

**Figure 12:**
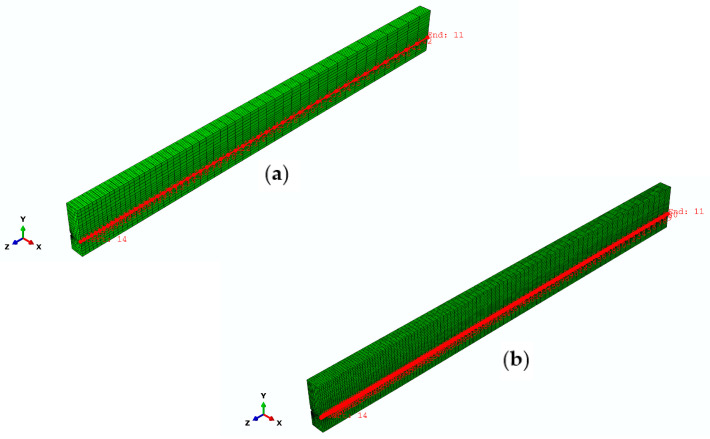
Nodes location on a concrete surface at the level of the FRP tendons in the finite element models: (**a**) FE model with course mesh; (**b**) FE model with fine mesh.

**Figure 13:**
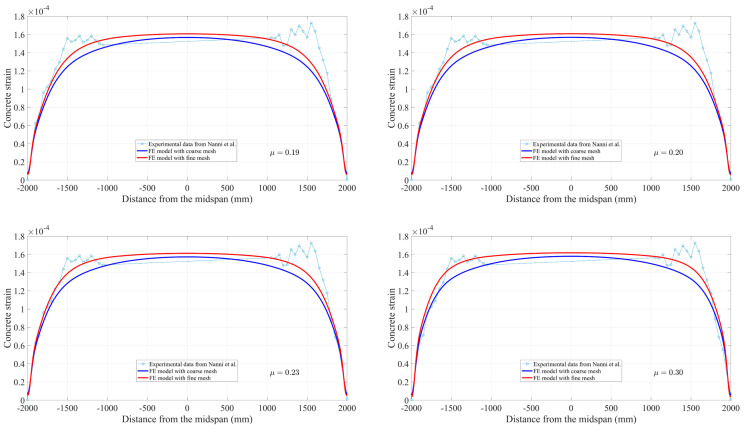
Comparison of the strain profile at 100% release.

**Figure 14:**
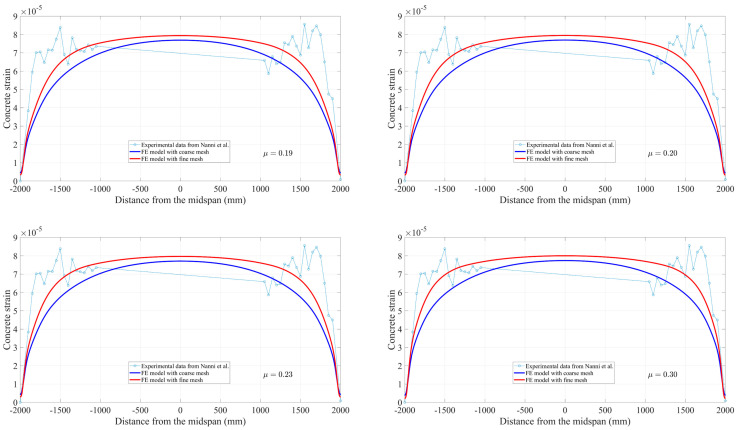
Comparison of the strain profile at 50% release.

To further demonstrate the mesh convergence and the feasibility and accuracy of the 3D FEA solution, a comparison is made between the FE results and the strain profile from the experiment [41]. Figure 12 represents a group of nodes that are located at the position on a concrete surface at the level of the FRP tendons within the fine FE model and coarse FE model, which provides the shear strain value along the beam, corresponding to the results recorded in the test. The values predicted by the FE model and the experimental results at 100% and 50% of prestress force release are compared in Figure 13 and Figure 14, respectively. As can be seen, there is a reasonable agreement with the strain measurements. Compared to the effective prestress strain of 153 microstrains collected through the test at 100% force release, the fine and coarse FE model approximately overestimates by 4%. Corresponding to the effective prestress strain of 67 microstrains at 50% force release, they are exceeded by approximately 15% to 19%.

Based on the 95% AMS method, comparisons of transfer length predicted by the present FE models with measured results [41] are summarized in Table 3. It can be clearly observed that the value of transfer length slightly decreases with the increase of the friction coefficient in both FE models, especially for the fine mesh model. The difference is due to the stronger bond that will shorten the distance to reach the effective prestress. Moreover, it is shown that the predictions using the FE model with a fine mesh match the experimental results better than those from the coarse mesh model through convergence analysis. The difference between the measured transfer length for high pretension and the fine mesh FE model with a friction coefficient of μ = 0.30 is 10%. Since the method for determining transfer length during the test is carried out by combining the 95% average maximum strain (AMS) method with the measurement of the strain change in the concrete. Inconsistency and preference may exist in the evaluation of the strain plateau between different researchers. For another reason, the measured values of transfer length reported in Table 3 are the average of multiple test results [41] in which FRP tendons are released on different days. Therefore, though a 10% difference between the transfer length predicted by the FE model and that from the experiment is not an ideal value, the feasibility of the present method through reasonable agreements between the measured strain profile and that predicted by the FE model is demonstrated. At the same time, it can be noticed that the FE model’s performances are not as good at a low pretension level of 50%; the reason will be analyzed and discussed in the later section.

## 5. Comparison and Discussion

**Figure 15:**
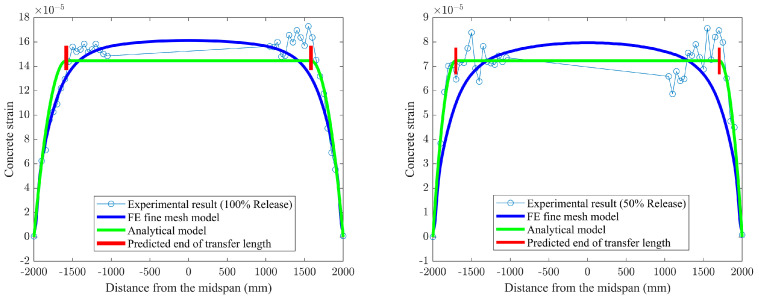
Strain profile predicted by finite element model at 100% and 50% release versus analytical solutions.

For further proving the performance of the application of composite beam theory on predicting the transfer length for prestressed FRP tendons strengthening pretensioned concrete members, the present FE model is used to compare with the previously developed analytical solutions. As can be seen from Figure 15, it is worth noting that theoretical results for 50% and 100% release levels are in excellent agreement with those from the experiment compared to the FE model’s result. In particular, the slope of both curves related to the rate of strain change within the transfer zones can be accurately calculated by the analytical model using composite beam theory. In the corresponding zones, a small discrepancy exists between the FE model for 50% release force and strain profile measurements, which fully demonstrates that determining the transfer length is mainly dependent upon the understanding and definition of bond behavior during the simulation process. This is also the reason why the FE fine model with μ = 0.3 significantly overestimates the values measured in the tests at 50% force release by 107%.

Since it is not possible to exactly match the values measured from testing, the local bond–slip relationship between the FRP tendons and the concrete is taken into account to predict the transfer length using closed-form solutions from Equation (26) in the analytical model. This results in an error between the predictions and the test values of 7% and 26% for high pretension (100% force release) and low pretension (50% force release), respectively. In this perspective, the accuracy of the transfer length obtained from the theoretical solution in terms of partially composite action is superior to that of the numerical simulation by using a Coulomb friction model based on FEA.

**Figure 16:**
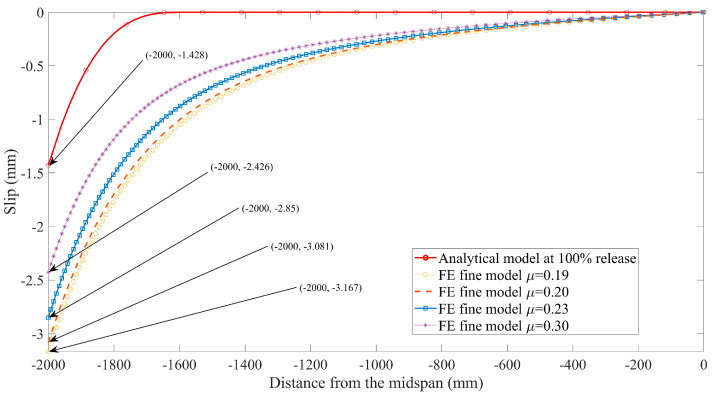
Slip predicted by finite element model at 100% release versus analytical solutions.

**Figure 17:**
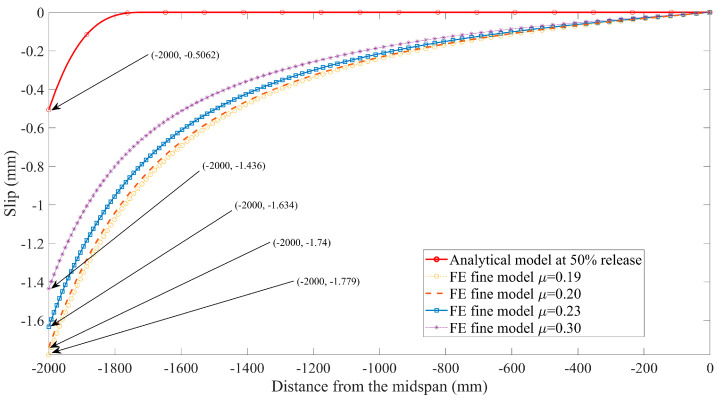
Slip predicted by finite element model at 50% release versus analytical solutions.

In addition, extensive studies [26,42] have shown that the influence of interface slip on the mechanical behavior of composite structures cannot be neglected. For this reason, the curves presented in Figure 16 and Figure 17 are used to conduct the comparison between the slip predicted by the closed-form solution given by Equation (25) and those from the FE models with various friction coefficients. Note that the value predicted by the analytical solution using composite beam theory is smaller than the FE model predictions; the main reason is attributed to different bond-behavior models that are adopted in theoretical and numerical solutions. The local bond stress–slip relationship τ = τ(s) used in the analytical model is based on the experimental investigation from the available literature [7], in which the effects of three factors on bond behavior are comprehensively considered, including chemical adhesive, friction, and mechanical interlocking, as mentioned in the previous section. Whereas only friction is modeled as a tangential behavior associated with the friction coefficient from the pullout test [31] during the process of FE simulation, many studies have confirmed that friction plays a major role in the interaction between FRP tendons and concrete. However, the interface slip can be still reduced by the other two factors, i.e., adhesion and mechanical interlocking. This is also the reason why the prediction values for transfer length from FE models are larger than the test results summarized in Table 3. To a certain extent, it is further proved that the analytical solution for FRP-strengthening concrete members, considering the empirical bond–slip relationship in terms of composite beam theory, is reasonable.

On the other hand, it is obvious to see that a remarkable increase in interface slip occurs when the value of the friction coefficient decreases as shown in Figure 16 and Figure 17. This is because the bond strength between concrete and FRP tendons is reduced as the decrease of the friction coefficient, resulting in the larger slip.

**Figure 18:**
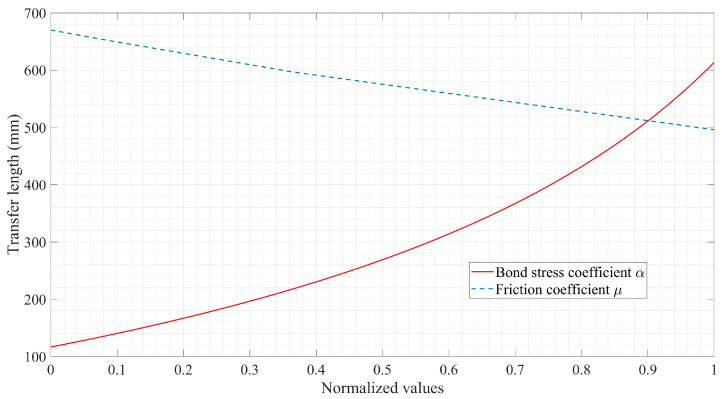
Influence of bond-stress coefficient α and friction coefficient μ on transfer length.

Through normalizing the parameters, the influence of the friction coefficient μ tabulated in Table 1 and the bond-stress coefficient α (α = 0~0.6) given by the expression of τ = τ(s) on the transfer length is compared. It has been found from the result, as represented in Figure 18, that the transfer length of prestressed FRP tendons in pretensioned concrete members is exponentially proportional to the bond-stress coefficient α and inversely proportional to the friction coefficient μ. Furthermore, from the perspective of the varying tendencies of the curves, the extent of the effect of the bond-stress coefficient is more distinctive than the friction coefficient. In essence, different forms of the function that describes the bond behavior are adopted in the theoretical solution and numerical simulation that lead to differing impacts.

In analytical solutions using composite beam theory, the BEP expression is chosen as the bond–slip relationship τ = τ(s) to estimate the transfer length of pretensioned concrete members prestressed with FRP tendons. An important difference from the linear equation modeling the interaction between contact bodies using FEM is that the function form of τ = τ(s) is a power function of the slip s in the analytical solution. By understanding the concept of transfer length, the distance that the effective prestressing force is transferred by the bond stress τ from the prestressed tendons to the concrete in which the axial force of concrete increases from zero to a constant. In other words, there is no interactive shear stress related to the bond between FRP tendons and concrete outside of the transmission zone. This exactly fits the typical characteristics of power functions with 0 < α < 1, where the slope of the curve gradually becomes flat as the variable increases. With the use of the BEP relationship to explain the bond mechanism, another advantage is that the closed-form solution for transfer length by means of Dirichlet and Neumann boundary conditions avoids many approximations and computational effort compared to the 95% AMS method in the numerical simulation.

## 6. Conclusions

Testing is considered to be the best way to predict a phenomenon and obtain necessary information. However, large-scale testing is time-consuming, expensive, and has many limitations. With this consideration, along with the need to further verify the accuracy and feasibility of the previously developed method, a three-dimensional FE model of pretensioned concrete members with prestressing FRP tendons was developed. Despite the numerous numerical research on reinforced concrete beams strengthened with conventional FRP bars, none of the existing studies considered the effects of friction coefficients on the transfer length. To bridge this gap in the literature, the different friction coefficients between FRP tendons and the surrounding concrete obtained by experimental studies are fully considered in this study. Based on the data reported from the pullout test, fine and coarse FE models were implemented for convergent analysis, respectively.

In addition, the general approach of composite beam theory, as the key innovation of this research, is derived for providing convenient use in engineering practice, specifically for FRP-strengthening concrete members. Lastly, a comparison between the analytical solution and the FE simulation is carried out and discussed. The main accomplishments and conclusions are as follows:

1. A general form of the governing equations has been presented specifically for FRP-strengthening concrete members in terms of composite beam theory. Associating with the knowledge of the local bond stress–slip relationship τ = τ(s) between FRP and concrete, the closed-form solution can be solved under corresponding boundary conditions;
2. Comparisons with the experimental data demonstrate good agreement, which indicates that the proposed FE model with fine mesh is acceptable. The measured transfer length for high pretension agrees with the prediction from the fine FE model with the friction coefficient α = 0.3 within a 10% range. The consistency between the FE model results and the previously developed analytical solutions demonstrate that theoretical results using composite beam theory are superior to that of the numerical simulation;
3. Although friction plays a key role in the interaction between concrete and prestressed tendons, the slip-prediction comparisons show that if the adhesion and mechanical interlocking are ignored, the bond behavior cannot be accurately evaluated;
4. The transfer-length prediction is strongly dependent on the adopted function form of the bond–slip relationship τ = τ(s) between the concrete and FRP tendons in the analytical model using composite beam theory. For the analytical solution of the mechanical behavior of concrete members strengthened with FRP in terms of partially composite action, the most critical issue is to have knowledge of the local bond–slip relationship in the interface region.

Therefore, it is necessary to adopt a new method to describe the bond behavior between concrete and FRP tendons in the future FE simulation. In this process, adhesion, friction, and mechanical interlocking must be fully considered in order to provide more accurate predictions and facilitate engineering applications.

## Figures and Tables

**Table 1:** Friction coefficient used for FRP tendons [31].

FRP Tendons	Concrete	Friction Coefficient
Vinylon FRP rods	HEM	0.23
Carbon FRP strands	HEM	0.30
Vinylon FRP rods	HEM and mortar	0.20
Carbon FRP strands	HEM and mortar	0.19

**Table 2:** Material parameters used for FE simulation [41].

Part	Modulus of Elasticity (MPa)	Poisson’s Ratio	Density (tonne/mm^3^)
Concrete	25,070	0.20	2.4 × 10^−9^
AFRP tendons	67,600	0.35	1.4 × 10^−9^

**Table 3:** Comparison of transfer length between FE simulation and experiment.

Beam Group	Measured (mm) [41]	Predicted by FE Model (mm)				
			μ = 0.19	μ = 0.20	μ = 0.23	μ = 0.30
High pretension (100% force release)	450	Fine	670	651	597	496
		Coarse	939	939	893	806
Low pretension (50% force release)	400	Fine	967	943	897	831
		Coarse	1192	1192	1192	1138

## Data Availability

The majority of data presented in this study are available upon reasonable requests to the corresponding author.
